# *Floresorchestia kongsemae* sp. n. a new species (Crustacea: Amphipoda: Talitridae) from Kasetsart University, Bangkok, Thailand

**DOI:** 10.3897/BDJ.9.e63197

**Published:** 2021-04-08

**Authors:** Anotai Suklom, Patchara Danaisawadi, Koraon Wongkamhaeng

**Affiliations:** 1 Department of Zoology, Faculty of Science, Kasetsart University, Bangkok, Thailand Department of Zoology, Faculty of Science, Kasetsart University Bangkok Thailand

**Keywords:** Land hopper, Talitridae, man-made pond, Bangkok

## Abstract

**Background:**

The genus *Floresorchestia* Bousfield, 1984 is widely distributed in terrestrial and marine coastal habitats. It has been found from coastal South Africa through to the Indo-Pacific region and the Caribbean Sea in Central America. Two species of *Floresorchestia* have been reported in Thailand, *Floresorchestia boonyanusithii* Wongkamhaeng et al. 2016 and *Floresorchestia buraphana* Wongkamhaeng et al. 2016. This work reports on a new species of *Floresorchestia* found at Kasetsart University in a man-made pond and neighbouring areas.

**New information:**

Classification of the new species was achieved by considering the left mandible 5-dentate; gnathopod 2 posterior margin merus carpus and propodus of gnathopod 2 covered in palmate setae, palm reaching about 33% along posterior margin; uropod 3 peduncle with three robust setae; telson dorsal mid-line half the length of its breadth and four robust setae per lobe.

## Introduction

*Floresorchestia*, a member of talitrid amphipods, has been reported in the Indo-Pacific Region. This genus contains members of 25 coastal (16) and terrestrial (9) species (Lowry and Springthorpe 2015, Lowry and Springthorpe 2019) which inhabit a variety of habitats, including supralittoral, mountain and moist areas near freshwater streams. Many reports have found that amphipods survive in man-made habitats, suggesting that they have good adaptation and high migration abilities (Friend and Richardson 1986, Lowry and Springthorpe 2015, Wongkamhaeng et al. 2016).

This paper reviews the genus *Floresorchestia* in Thailand and describes a new species from a man-made habitat at Kasetsart University, Bangkok. The dichotomous key in this region are provided.

## Materials and methods

This study is based on material collected in February 2020 from the leaf litter of a man-made swamp in front of Chakrabandhu Pensiri Hall, Kasetsart University, central Thailand. Samples were collected using a hand-net and carefully transferred into a plastic container for fixation in 10% buffered formalin. In the laboratory, amphipod specimens were sorted and stored in 70% alcohol. The animals were then examined under a stereomicroscope and later selected for dissection. The dissected specimens' appendages were examined and figures were produced using a camera lucida attached to an Olympus CH30 light microscope. Pencil drawings were scanned and digitally inked using a WACOM Bamboo CTH-970 graphics board following the method described by Coleman (2003). Setal and mouthpart classifications were made following Zimmer et al. (2009).

**Figure 1.**
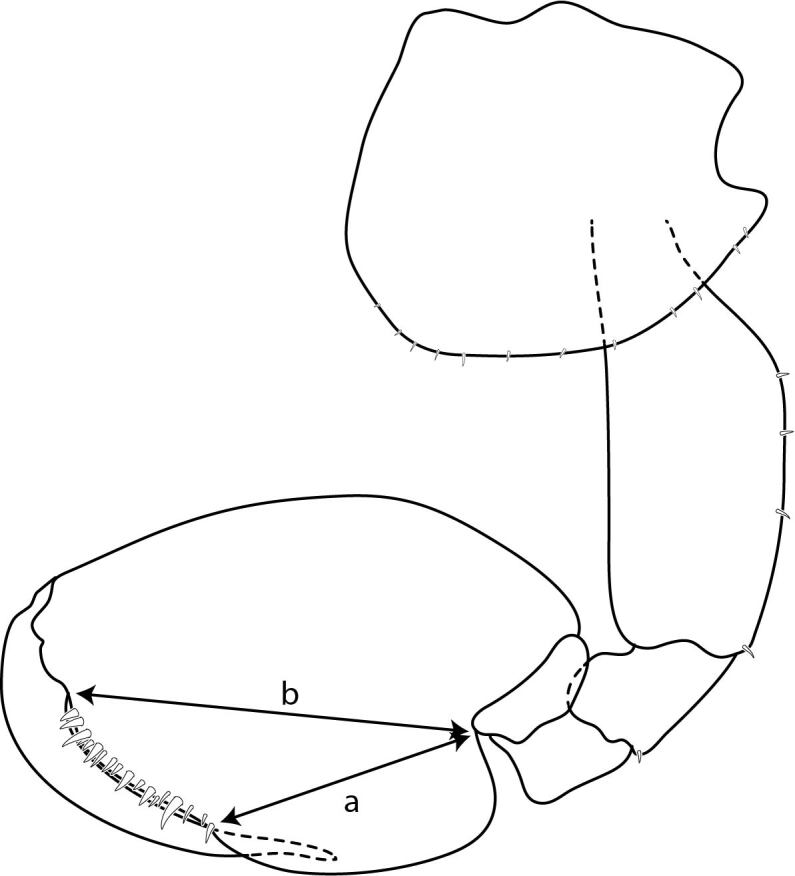
The measurement method for the length of male gnathopod 2 palm and posterior margin of propodus.

The palm measurement length was made following Lowry and Springthorpe (2015) as a percentage of the length of the propodus of male gnathopod 2. The percentage is calculated using the formula 100(1 - a/b)% (Fig. 1) where ‘a’ is the length of the posterior margin measured from the seta at the corner of the palm to the base of the propodus and ‘b’ is the length of the propodus measured from the base of the dactylus to the base of the propodus.

**Repository**

THNHM-Iv = Thailand Natural History Museum, Thailand.

Figure legend: A, antenna; G, gnathopod; HD, head; LL, lower lip; MD, mandible; MX, maxilla; MP, maxilliped; P, pereopod; Pl, pleopod; T, telson; U, uropod; UR, urosome; UL, upper lip; R, right; L, left; ♂, male; ♀, female.

## Taxon treatments

### Floresorchestia kongsemae sp. n.

87CD0E76-E0AA-569D-A2DD-C7D84C9AAFBE

FEDEE681-763B-42F8-819A-033FF8F575AD

#### Materials

- **Type status:** Holotype. **Occurrence:** catalogNumber: THNHM-Iv-18766; recordedBy: Koraon Wongkamhaeng; individualCount: 1; sex: male; lifeStage: adult; occurrenceID: F398AF06EE6EC91745491C1A2F97AE85.mc.DBE012D8755E2F68B6DE3633B88A2265; **Taxon:** scientificName: Floresorchestia kongsemae; **Location:** country: Thailand; stateProvince: Bangkok; locality: Kasetsart University, Lat Yao, Chatuchak; verbatimElevation: 0 m; locationRemarks: Chakrabandhu Pensiri Hall, Kasetsart University; 0 m, 13°51'00.8"N 100°34'12.3"E, 2020.02.01, Pitfall trap; verbatimCoordinates: 13°51'00.8"N 100°34'12.3"E; decimalLatitude: 13.850222; decimalLongitude: 100.570083; georeferenceProtocol: GPS; **Identification:** identifiedBy: Anotai Suklom; dateIdentified: 2020; **Event:** samplingProtocol: Pitfall trap; eventDate: 02/01/2020; **Record Level:** language: en; collectionCode: Crustaceans; basisOfRecord: slide
- **Type status:** Other material. **Occurrence:** catalogNumber: THNHM-Iv-18767; recordedBy: Koraon Wongkamhaeng; individualCount: 1; sex: female; lifeStage: adult; occurrenceID: F398AF06EE6EC91745491C1A2F97AE85.mc.376CF07F91D0945EF037940C85C70F0B; **Taxon:** scientificName: Floresorchestia kongsemae; **Location:** country: Thailand; stateProvince: Bangkok; locality: Kasetsart University, Lat Yao, Chatuchak; verbatimElevation: 0 m; locationRemarks: Chakrabandhu Pensiri Hall, Kasetsart University; [0 m, 13°51'00.8"N 100°34'12.3"E, 2020.02.01, Pitfall trap; verbatimCoordinates: 13°51'00.8"N 100°34'12.3"E; decimalLatitude: 13.850222; decimalLongitude: 100.570083; georeferenceProtocol: GPS; **Identification:** identifiedBy: Anotai Suklom; dateIdentified: 2020; **Event:** samplingProtocol: Pitfall trap; eventDate: 02/01/2020; **Record Level:** language: en; collectionCode: Crustaceans; basisOfRecord: slide
- **Type status:** Paratype. **Occurrence:** catalogNumber: THNHM-Iv-18768; recordedBy: Koraon Wongkamhaeng; individualCount: 15; sex: female; lifeStage: adult; occurrenceID: F398AF06EE6EC91745491C1A2F97AE85.mc.737282B8E1D92C8671898323582290D1; **Taxon:** scientificName: Floresorchestia kongsemae; **Location:** country: Thailand; stateProvince: Bangkok; locality: Kasetsart University, Lat Yao, Chatuchak; verbatimElevation: 0 m; locationRemarks: Chakrabandhu Pensiri Hall, Kasetsart University; [0 m, 13°51'00.8"N 100°34'12.3"E, 2020.02.01, Pitfall trap; verbatimCoordinates: 13°51'00.8"N 100°34'12.3"E; decimalLatitude: 13.850222; decimalLongitude: 100.570083; georeferenceProtocol: GPS; **Identification:** identifiedBy: Anotai Suklom; dateIdentified: 2020; **Event:** samplingProtocol: Pitfall trap; eventDate: 02/01/2020; **Record Level:** language: en; collectionCode: Crustaceans; basisOfRecord: PreservedSpecimen

#### Description

Based on holotype male, 5.5 mm, THNHM-Iv-18766

**Figure 2.**
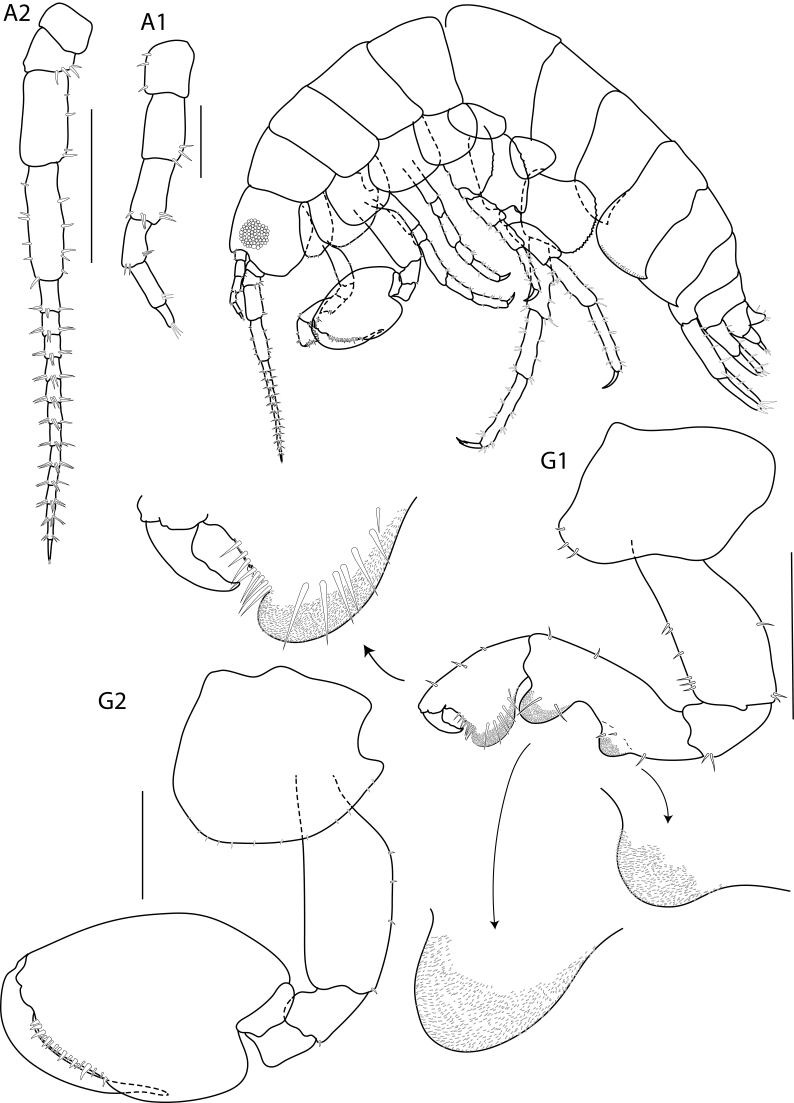
*Floresorchestia kongsemae* sp. n. holotype, male, 5.5 mm (THNHM-Iv-18766), Kasetsart University, Bangkok. Scale for A1, G1 and G2 represent 0.5 mm and A1 represents 0.2 mm.

**Head**. (Fig. 2) Eye large (greater than 1/3 head length). Antenna 1 short, exceeding article 4 of antenna 2 peduncle. Antenna 2 less than half body length, peduncular article slender; article 2 and 3 shortest; article 5 longer than article 4; flagellum of 13 articles and subequal to peduncle, final article of flagellum with an apical cluster of setae.

**Figure 3.**
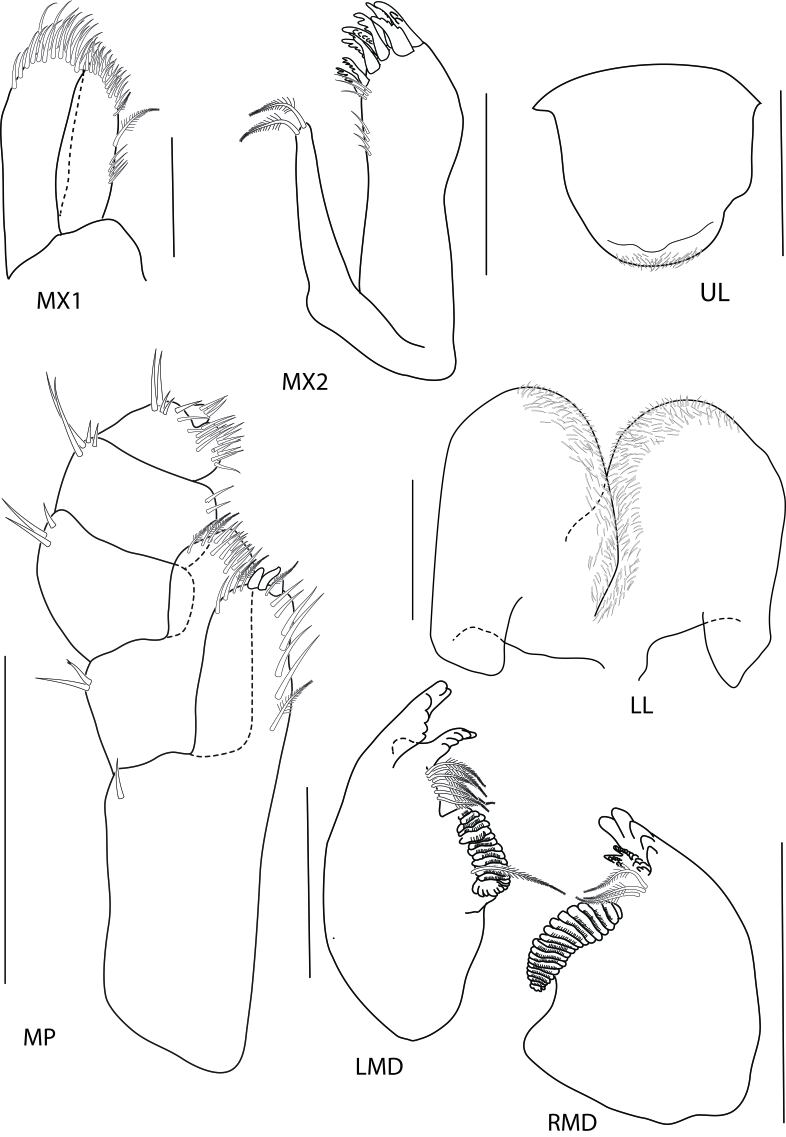
*Floresorchestia kongsemae* sp. n. holotype, male, 5.5 mm (THNHM-Iv-18766), Kasetsart University, Bangkok. Scale for MX1, MX2, MP, LMD and RMD represents 0.2 mm and UL and LL represents 0.1 mm.

Upper lip (Fig. 3) broad, deep, apex rounded. Left mandible incisor 6-dentate, lacinia mobilis 5-dentate, molar strong. Right mandible incisor 6-toothed, lacinia mobilis 16-dentate, molar process strong, with 19—20 striate. Maxilla 1 inner plate slender with two terminals plumose setae; the outer plate without palp, with nine robust serrated setae. Maxilla 2 inner plate narrow, slightly shorter than outer, with ca. 19 subapical robust setae, one plumose robust seta at inner corner; outer plate with ca. 27 subapical robust setae more or less in 2 rows. Maxilliped inner plate with apical and subapical plumose setae and three large conical robust setae; outer plate with subapical and plumose setae, two rows and plumose setae; palp article 2 distromedial lobe well developed; article 4 reduced, button-shaped.

**Figure 4.**
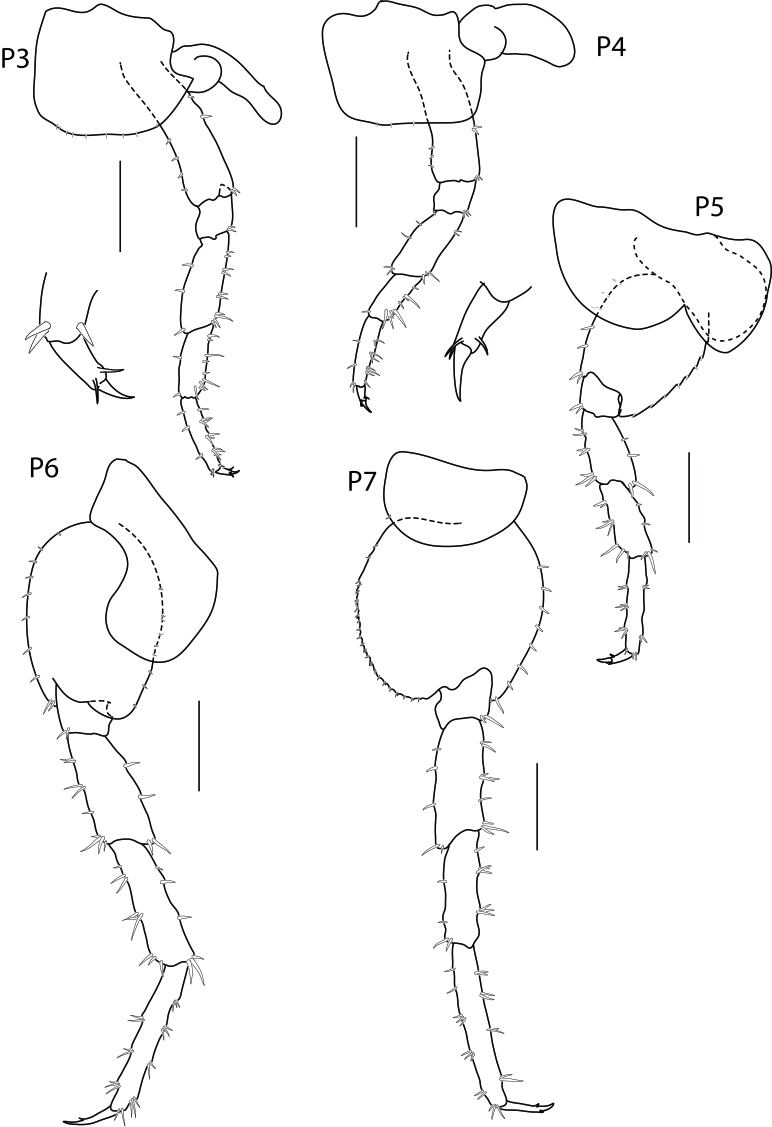
*Floresorchestia kongsemae* sp. n. holotype, male, 5.5 mm (THNHM-Iv-18766), Kasetsart University, Bangkok. All scale bars represent 0.5 mm.

**Pereon.** (Fig. 4) Gnathopod 1 sexually dimorphic; subchelate in male, coxa smaller than coxa 2, ventral margin with three robust setae, anterior margin straight, posterior side rounded; basis pararell, anterior margin with two setae, posterior margin with three setae; carpus and propodus each with lobe covered with robust setae; carpus anterodistal corner with two robust setae, posterior margin with three robust setae; propodus subtriangular with well development posterior lobe, anterior side with two groups of robust setae each, posterior margin with five robust setae; dactylus subequal in length to palm.

Gnathopod 2 (Fig. 2) subchelate, sexually dimorphic; basis slightly pararell, posteriorly, posterior margin with 3 robust setae; ischium with rounded lobe on anterodistal margin, carpus and propodus without posterior lobe; carpus reduced (enclosed by merus and propodus); propodus, anterior margin naked, palm lined with 17 robust setae; dactylus longer than palm, attenuated distally.

Pereopod 3 (Fig. 4) coxa longer than broad, with posterior process; merus longer than carpus and propodus, slightly expanded; posterior margin lined with group of robust setae; carpus shortest, posterior margin with group of robust setae; propodus slender and longer than carpus; dactylus without notch on posterior margin.

Pereopod 4 (Fig. 4) slightly shorter than pereopod 3; coxa longer than broad, without posterior process; merus and carpus shorter than those of pereopod 3; merus distally expanded, longer than carpus or propodus; carpus shorter than propodus posterior margin with group of robust setae; propodus slender with group of robust setae in posterior side; dactylus slender and longer than dactylus 3, with a notch in posterior margin.

Pereopod 5 (Fig. 4) coxa bilobed, anterior lobe more distinct than posterior lobe; basis posterior margin serrate and small setae; merus and carpus distally expanded, propodus distinctly slender, longer than merus or carpus.

Pereopod 6 (Fig. 4) coxa bilobed, ventral margin perpendicular to anterior margin; merus distally expanded, anterior margin with group of robust setae; carpus expanded less than merus, anterior margin lined with 3 groups of robust setae; propodus slender, longer than merus or carpus.

Pereopod 7 (Fig. 4) coxa reduced; posterior margin of basis with distinct minute serrate with small setae; propodus longer than merus or carpus; dactylus short.

**Figure 5.**
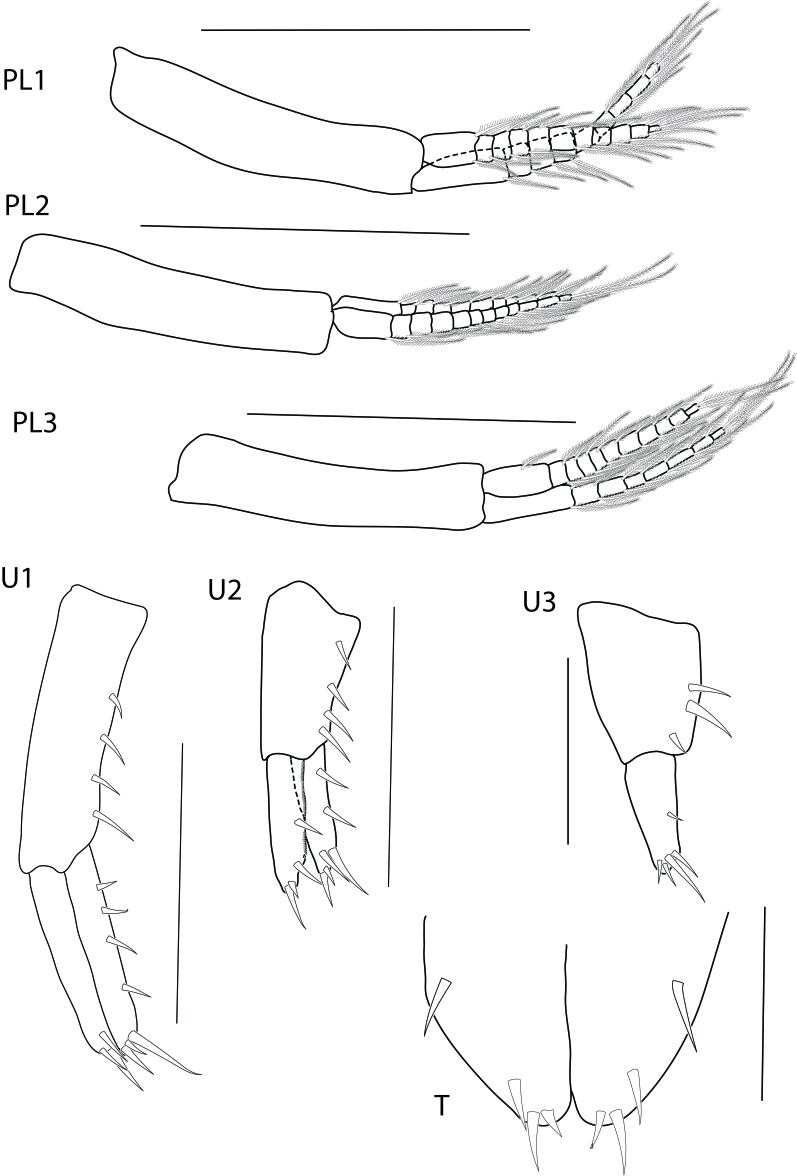
*Floresorchestia kongsemae* sp. n. holotype, male, 5.5 mm (THNHM-Iv-18766), Kasetsart University, Bangkok. Scale for PL1-3 represents 0.5 mm, U1-2 represent 0.2 mm and U3 and T represent 0.1 mm.

**Pleon.** (Fig. 5) all pleopods well developed, biramous. Pleopod 1 peduncle without setae, inner ramus subequal to outer ramus, shorter than peduncle, with nine articles; outer ramus with eleven articles. Pleopod 2 peduncle without setae, inner ramus with ten articles; outer ramus with twelve articles, both rami shorter than peduncle. Pleopod 3 peduncle without setae; inner ramus subequal to outer ramus, shorter than peduncle; inner ramus with eight articles; outer ramus with eleven articles.

Epimera vertical slits present on plates 2 and 3. Epimera 2 with 23 slits. Epimera 3 with 16 slits. Epimeron 2 subequal in length to epimeron 3. Epimeron 3 ventral margin smooth, posteroventral corner smooth without setae.

Uropod 1 (Fig. 5) peduncle with eight robust setae; inner ramus subequal in length to outer ramus; inner ramus with one row of robust setae, with four robust setae; outer ramus naked. Uropod 2 peduncle with four robust setae; inner ramus subequal in length to outer ramus, with two marginal robust setae; outer ramus with two marginal robust setae. Uropod 3 uniramus, peduncle with three robust setae; ramus subequal to peduncle, with one marginal robust setae and four apical robust setae.

Telson (Fig. 5) longer than broad, apically cleft, dorsal mid-line half of the telson, with four marginal and apical robust setae per lobe.

**Female (Sexually dimorphic characters)**

**Based on allotype female 8.8 mm**,THNHM-Iv-18767

**Figure 6.**
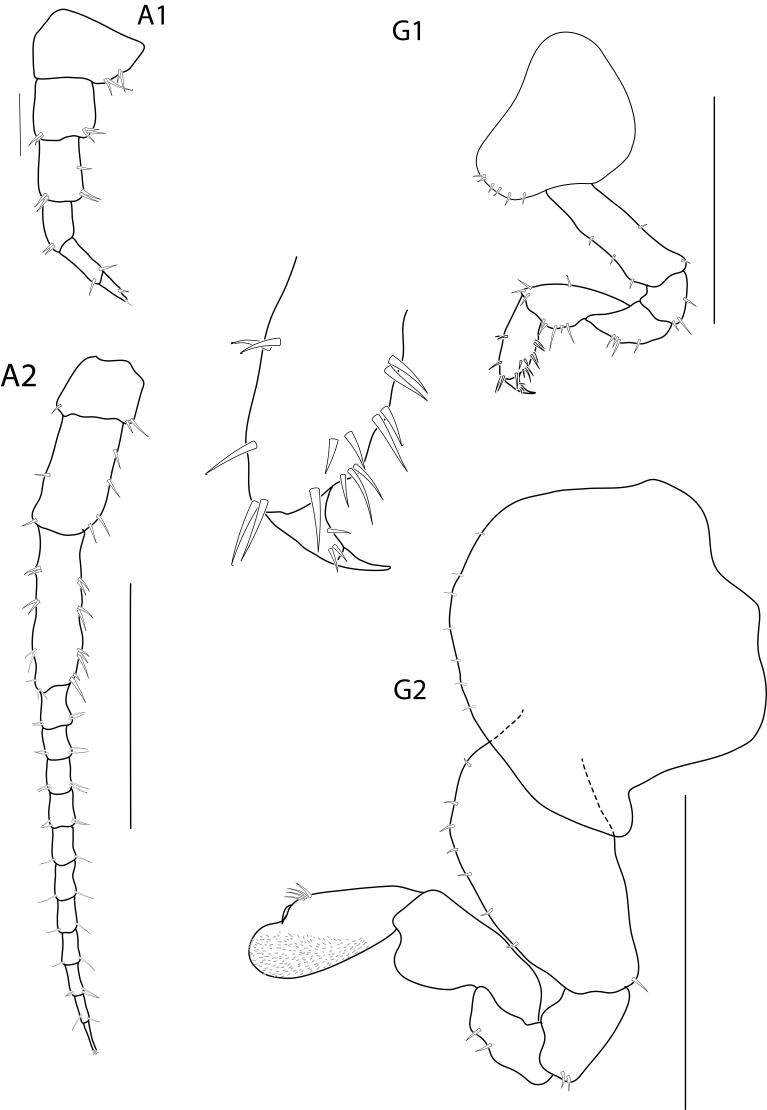
*Floresorchestia kongsemae* sp. n. allotype, female 8.8 mm (THNHM-Iv-18767), Kasetsart University, Bangkok. Scale bars for A2, G1, and G2 represent 0.5 mm and A1 represents 0.2 mm.

**Head.** (Fig. 6) Antenna 2 less than half body length, peduncular articles slender; articles 2, 3 shortest; article 5 longer than article 4; flagellum of 11 article and longer than peduncle. Left mandible incisor 5-dentate, lacinia mobilis 5-dentate.

**Pereon.** (Fig. 6) Gnathopod 1 coxa anterior margin straight, ventral margin with robust setae; merus subtriangular, posterior margin without lobe; carpus anterior margin with one robust setae, posterior margin with three robust setae covering the lobe; propodus anterior margin with two groups of robust setae; dactylus posterior margin with three robust setae.

Gnathopod 2 mitten-shaped; coxa along anterior margin with robust setae; basis distinctly expanded, anterior margin with robust setae, posteroventral margin with one robust setae; posterior margin of carpus and propodus with lobe cover in palmate setae; carpus posterior lobe well developed and obtuse; propodus anterior margin near dactylus with four robust setae, ventral margin with setae.

##### Remarks

*Floresorchestia kongsemae* sp. nov. is the third member in the genus *Floresorchestia* that have been reported in Thailand. *Floresorchestia kongsemae* sp. n. differs from *F. boonyanusithii* in the following ways: 1) the left mandible lacinia mobilis has 5-dentate (vs. 4-dentate); 2) uropod 1 peduncle with eight robust setae and three robust marginal setae in inner ramus (vs. four robust setae in the peduncle, four robust marginal setae in inner ramus); 3) uropod 2 peduncle with four robust marginal setae, outer ramus with two robust marginal setae (vs. peduncle with three robust setae, outer ramus with two robust marginal setae); 4) uropod 3 peduncle with three robust setae, apical four robust setae (vs. peduncle with two robust setae, apical three robust setae) (Table 1).

*Floresorchestia kongsemae* sp. n. is closely related to *F. buraphana*, a beach-hopper found in a freshwater swamp at Burapha University. Both species having: 1) left mandible lacinia mobilis 5-dentate; 2) gnathopod 1 merus, carpus and propodus each with palmate lobe; 3) uropod 1 inner ramus with three robust marginal setae, outer ramus without robust marginal setae; 4) uropod 2 peduncle with four robust setae, outer ramus with two robust marginal setae and 5) uropod 3 ramus with one robust marginal setae. *F. kongsemae* can be distinguished from *F. buraphana* in the left mandible incisor with 6 teeth (5 teeth in *F. buraphana*), maxilla 1 without palp (with palp in *F. buraphana*), uropod 1 peduncle with eight robust setae (with nine robust setae in *F. buraphana*), uropod 3 peduncle with three robust setae, apical ramus with four robust setae (vs. with two robust setae in the peduncle, apical ramus with three robust setae) and telson with four robust setae per lobe (vs. with five robust setae per lobe).

#### Etymology

The species is named in honour of Dr. Mesayamas Kongsema of Kasetsart University, Thailand, who contributed to the study of the life history of this new species.

#### Distribution

Bangkok

#### Ecology

*Floresorchestia kongsemae sp. n.* is found in man-made ponds, similar to other terrestrial species found in moist areas or areas covered by organic materials. The amphipods generally live on the surface (1-2 inches under the surface) during the rainy season or optimal weather days (25-30°C). During sunny days or during periods of high temperature (> 30°C), they burrow deeper looking for more humid conditions.

#### Biology

*Floresorchestia kongsemae sp. n.* has two breeding periods per year (late summer/early rainy season and the end of the rainy season).

## Identification Keys

### Key to determination of Thai species of *Floresorchestia*

**Table:** 

1	Left mandible lacinia mobilis 4-dentate; uropod 3 ramus without marginal robust setae; telson with four robust setae per lobe	*Floresorchestia boonyanusithii*
–	Left mandible lacinia mobilis 5-dentate; uropod 3 ramus with marginal robust setae	2
2	Left mandible incisor with 6 teeth; maxilla 1 without palp; Uropod 3 peduncle with three robust setae, ramus with or without marginal setae; telson with four robust setae per lobe	*F. kongsemae* sp. n.
–	Left mandible incisor with 5 teeth; maxilla 1 with palp; Uropod 3 peduncle with two robust setae, ramus with a marginal robust seta; telson with five robust setae per lobe.	*F. buraphana*

## Discussion

The new species identified in this study can confidently be assigned to the genus *Floresorchestia*, based on the combination of characters mentioned by Lowry and Springthorpe (2015) and Lowry and Springthorpe (2019): 1) vertical slits on the ventral margins of the epimera; 2) posterior margin of merus, carpus and propodus of gnathopod 1, each with lobe covered in palmate setae. *Floresorchestia*, as a speciose and wide-spread genus, is also diversified in terms of ecology, including 16 coastal and 9 terrestrial species (Lowry and Springthorpe 2019). In Thailand, *Floresorchestia kongsemae* sp. n. is the third known member in this genus. Other species are *Floresorchestia boonyanusithii*, a field-hopper found in north-eastern Thailand and *Floresorchestia buraphana*, a beach-hopper found in supralittoral man-made ponds located in eastern Thailand (Wongkamhaeng et al. 2016). In this study, samples of *Floresorchestia kongsemae* sp. n. were collected from a man-made pond in front of Chakrabandhu Pensiri Hall, Kasetsart University. *Floresorchestia kongsemae* is classified as a field-hopper and is present in coastal vegetation, meadows, pastures, grasslands and urban gardens, as well as amongst litter bands under stones and logs (Lowry and Myers 2019).

## Supplementary Material

XML Treatment for Floresorchestia kongsemae

## Figures and Tables

**Table 1.** Summary of diagnostic characters of *Floresorchestia kongsemae* and closely related species.

Species	Left mandible lacinia mobilis	Male gnathopod 1	Male gnathopod 2	Uropod 1	Uropod 3	Slit on epimera 2 and 3	telson
*Floresorchestia kongsemae* sp. n.	5-dentate	Palmate lobe on merus, carpus and propodus each covered in palmate setae	Palm reaching about 33% along posterior margin	Inner ramus with 3 marginal robust setae	Peduncle with 3 robust setae, rami with or without marginal setae	23 and 16 slits	4 robust setae per lobe
*Floresorchestia boonyanusithii* Wongkamhaeng, Dumrongrojwattana & Pattaratumrong, 2016	4-dentate	Palmate lobe on merus, carpus and propodus each covered in palmate setae	Palm extending between 31-35% along posterior margin	Inner ramus with 4 marginal robust setae	Peduncle with 2 robust setae, rami without marginal setae	27 and 20 slits	4 robust setae per lobe
*Floresorchestia buraphana* Wongkamhaeng, Dumrongrojwattana & Pattaratumrong, 2016	5-dentate	Palmate lobe on merus, carpus and propodus each covered in palmate setae	Palm extending between 36-40% along posterior margin	Inner ramus with 3 marginal robust setae	Peduncle with 2 robust setae, rami without marginal setae	25 and 15 slits	5 robust setae per lobe
*Floresorchestia hanoiensis* Hou & Li, 2003	4-dentate	Palmate lobe on merus, carpus and propodus each covered in palmate setae	Palm reaching about 38% along posterior margin	-	Peduncle with 2 robust setae, rami without marginal setae	22 and 5 slits	3 robust setae per lobe
*Floresorchestia kalili* Lowry & Springthorpe 2015	3 or 4-dentate	Palmate lobe on merus, carpus and propodus each covered in palmate setae	Palm reaching about 32% along posterior margin	Inner ramus with 5 marginal robust setae	Peduncle with 1 robust setae, rami with 1 marginal setae (♂)	26 and 19 slits	6-7 robust setae per lobe
*Floresorchestia malayensis* (Tattersal,1992)	4-dentate	Palmate lobe on merus, carpus and propodus each covered in palmate setae	Palm reaching about 30% along posterior margin	Inner ramus with 4 marginal robust setae	Peduncle with 1-2 robust setae, rami without marginal setae	33 and 27 slits	4 robust setae per lobe
*Floresorchestia thienemanni* (Schellenberg, 1931)	4-dentate	Palmate lobe on merus, carpus and propodus each covered in palmate setae	Palm reaching about 32% along posterior margin	Inner ramus with 4 marginal robust setae	Peduncle with 4-6 robust setae, rami without marginal setae	14 and 7 slits	7-8 robust setae per lobe
*Floresorchestia yehyuensis* Miyamoto & Morino, 2008	4-dentate	Palmate lobe on merus, carpus and propodus each covered in palmate setae	Palm reaching about 38% along posterior margin	-	Peduncle with 3 robust setae, rami with 1 marginal setae	33 and 15 slits	5 robust setae per lobe
*Gazia samroiyodensis* Azman, Wongkamhaeng & Dumrongrojwattana 2014	Male 4-dentate, female 6-dentate	Posterior lobe on carpus and propodus each covered in palmate setae	Palm reaching about 32% along posterior margin	Inner ramus with 4 marginal robust setae	Peduncle with 2 robust setae, rami with 2 marginal setae	21 and 13 slits	5 robust setae per lobe
